# Chest X-ray as a screening tool for blunt thoracic trauma in children

**DOI:** 10.1186/1757-7241-21-S1-S8

**Published:** 2013-05-28

**Authors:** NL Yanchar, K Woo, M Brennan, C Palmer, M Ee, J Crameri, B Sweeney

## Background

The role of computed tomography (CT) in paediatric blunt thoracic trauma (BTT) has not been adequately defined. However, thoracic CT is increasingly being used as a screening tool in paediatric trauma, and this represents a potential radiation risk to young patients.[1] We asked whether chest x-ray (CXR) findings in addition to other clinical and epidemiologic variables could be used to predict significant thoracic injuries, to inform the selective use of CT in paediatric BTT. We also wished to determine if these were discrepant from factors associated with the decision to obtain a thoracic CT in a cohort of injured patients.

## Methods

Data from three Level I paediatric trauma centres between April 1999 and March 2008 were reviewed retrospectively. Radiological and epidemiological data were collected. Logistic regression modeling was used to determine adjusted odds ratios for pre-CT findings associated with a CT diagnosis of any thoracic injury or a significant thoracic injury, as well as the decision to obtain a thoracic CT.

## Results

In our cohort of 426 patients, 175 (41.1%) had a significant chest injury, 207 (48.6%) had non-significant chest injury, and 44 (10.3%) had no injury at all. In total, 412 (96.7%) patients had an initial CXR upon admission to the trauma centre or referring centre emergency department. The presence of hydro- and/or pneumothorax on CXR significantly increased the likelihood of significant chest injury diagnosed on CT (OR 9.8; 95% CI 5.9-162) , as did the presence of subcutaneous emphysema (OR 18.1; 95% CI 2.1-154). A completely normal CXR was associated with a reduced risk of having significant thoracic injury (OR 0.33; 95% CI 0.19-0.56). The decision to obtain a thoracic CT, however, was only associated with later time period in the study and obtaining a CT scan of another body region.

## Conclusions

CXR can be used to screen for significant thoracic injuries and direct the selective use of thoracic CT in paediatric BTT. Further prospective studies are needed to validate these findings and develop guidelines that include CXR to define indications for thoracic CT in paediatric BTT.
